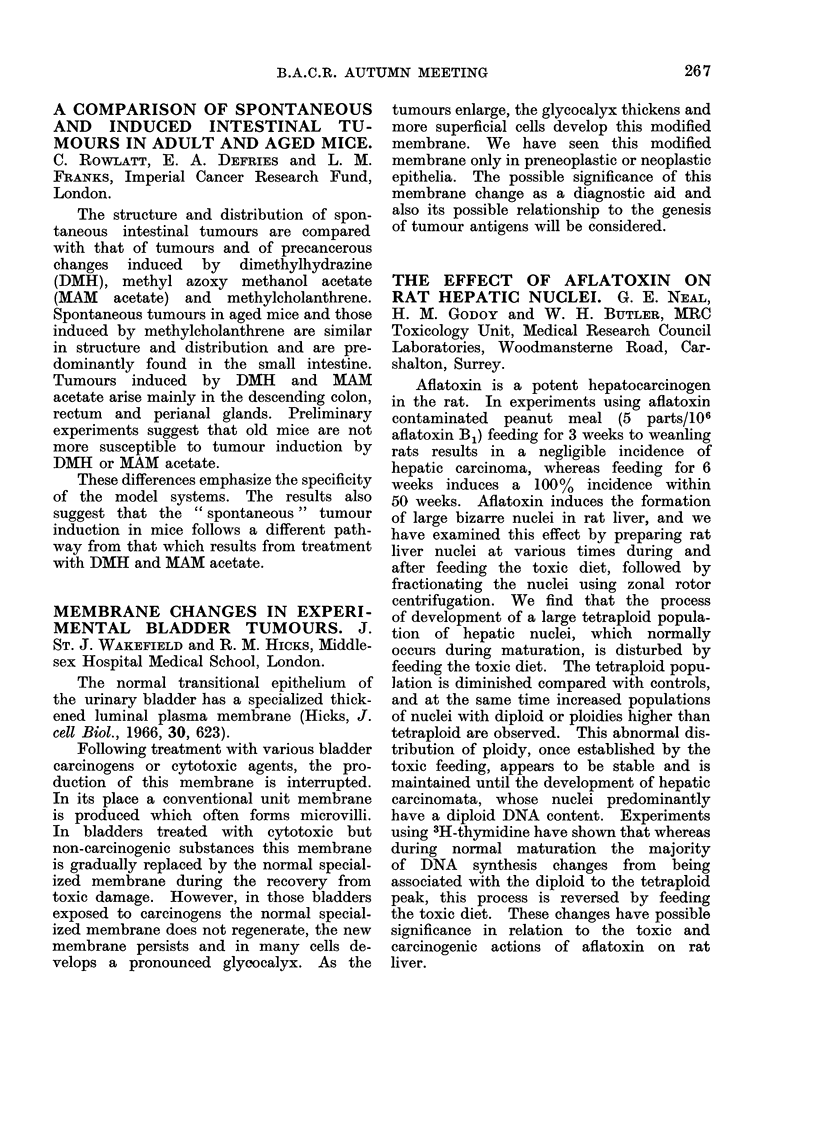# Proceedings: The effect of aflatoxin on rat hepatic nuclei.

**DOI:** 10.1038/bjc.1975.61

**Published:** 1975-02

**Authors:** G. E. Neal, H. M. Godoy, W. H. Butler


### THE EFFECT OF AFLATOXIN ON RAT HEPATIC NUCLEI.

G. E. NEAL, H. M. GODOY and W. H. BUTLER, MRC Toxicology Unit, Medical Research Council Laboratories, Woodmansterne Road, Carshalton, Surrey.

Aflatoxin is a potent hepatocarcinogen in the rat. In experiments using aflatoxin contaminated peanut meal (5 parts/$10^6$ aflatoxin $B_1$) feeding for 3 weeks to weanling rats results in a negligible incidence of hepatic carcinoma, whereas feeding for 6 weeks induces a 100% incidence within 50 weeks. Aflatoxin induces the formation of large bizarre nuclei in rat liver, and we have examined this effect by preparing rat liver nuclei at various times during and after feeding the toxic diet, followed by fractionating the nuclei using zonal rotor centrifugation. We find that the process of development of a large tetraploid population of hepatic nuclei, which normally occurs during maturation, is disturbed by feeding the toxic diet. The tetraploid population is diminished compared with controls, and at the same time increased populations of nuclei with diploid or ploidies higher than tetraploid are observed. This abnormal distribution of ploidy, once established by the toxic feeding, appears to be stable and is maintained until the development of hepatic carcinomata, whose nuclei predominantly have a diploid DNA content. Experiments using $^3$H-thymidine have shown that whereas during normal maturation the majority of DNA synthesis changes from being associated with the diploid to the tetraploid peak, this process is reversed by feeding the toxic diet. These changes have possible significance in relation to the toxic and carcinogenic actions of aflatoxin on rat liver.